# Role of human rhinovirus in triggering human airway epithelial-mesenchymal transition

**DOI:** 10.1186/s12931-017-0595-9

**Published:** 2017-05-30

**Authors:** Danielle M. Minor, David Proud

**Affiliations:** 1 Department of Physiology & Pharmacology, Snyder Institute for Chronic Diseases, Cumming School of Medicine, University of Calgary, Calgary, Alberta Canada; 2 University of Calgary, Faculty of Medicine, HRIC 4C50-54, 3280 Hospital Drive N.W., Calgary, AB T2N 4Z6 Canada; 3 Department of Physiology & Pharmacology, HRIC 4AC60, University of Calgary Cumming School of Medicine, 3280 Hospital Drive NW, Calgary, Alberta T2N 4Z6 Canada

**Keywords:** Human rhinovirus, Epithelial-mesenchymal transition, Transforming growth factor-β1, E-cadherin, Fibronectin, MAP kinases, SLUG

## Abstract

**Background:**

Structural changes in the airways, collectively referred to as airway remodeling, are a characteristic feature of asthma, and are now known to begin in early life. Human rhinovirus (HRV)-induced wheezing illnesses during early life are a potential inciting stimulus for remodeling. Increased deposition of matrix proteins causes thickening of the lamina reticularis, which is a well-recognized component of airway remodeling. Increased matrix protein deposition is believed to be due to the presence of increased numbers of activated mesenchymal cells (fibroblasts/myofibroblasts) in the subepithelial region of asthmatic airways. The origin of these increased mesenchymal cells is not clear, but one potential contributor is the process of epithelial-mesenchymal transition (EMT). We hypothesized that HRV infection may help to induce EMT.

**Methods:**

We used the BEAS-2B human bronchial epithelial cells line, which uniformly expresses the major group HRV receptor, to examine the effects of stimulation with HRV alone, transforming growth factor-β1 (TGF-β1), alone, and the combination, on induction of changes consistent with EMT. Western blotting was used to examine expression of epithelial and mesenchymal phenotypic marker proteins and selected signaling molecules. Cell morphology was also examined.

**Results:**

In this study, we show that two different strains of HRV, which use two different cellular receptors, are each capable of triggering phenotypic changes consistent with EMT. Moreover, both HRV serotypes synergistically induced changes consistent with EMT when used in the presence of TGF-β1. Morphological changes were also most pronounced with the combination of HRV and TGF-β1. Viral replication was not essential for phenotypic changes. The synergistic interactions between HRV and TGF-β1 were mediated, at least in part, via activation of mitogen activated protein kinase pathways, and via induction of the transcription factor SLUG.

**Conclusions:**

These data support a role for HRV in the induction of EMT, which may contribute to matrix protein deposition and thickening of the lamina reticularis in airways of patients with asthma.

## Background

The airways of patients with asthma display a number of characteristic structural changes that are collectively referred to as airway remodeling. These structural changes include an increase in airway smooth muscle mass, goblet cell hyperplasia/metaplasia leading to increased mucus production, epithelial fragility [1, 2], and increased numbers of fibroblasts and myofibroblasts in the subepithelial region that are associated with increased extracellular matrix protein deposition and thickening of the lamina reticularis [3, 4].

Although it was long thought that airway remodeling developed only after years of disease expression, bronchial biopsy studies have now established that components of airway remodeling can be demonstrated in early childhood, even prior to the formal diagnosis of asthma being made [5]. Several studies have demonstrated features of airway remodeling, including thickening of the lamina reticularis, increased smooth muscle mass, increased mucus gland area and angiogenesis in pre-school age children [6–9]. Interestingly, such features were not observed in symptomatic infants with reversible airflow obstruction [10]. This suggests that airway remodeling occurs in response to some inciting stimulus (or stimuli) in early life, and is not a congenital phenomenon.

Human rhinovirus (HRV) infections in early life are a common trigger for childhood wheezing illnesses [11, 12], and HRV-induced wheezing illnesses in the first 3 years of life are a major risk factor for subsequent development of asthma [13]. Longitudinal analysis has shown that pre-school age children have about six HRV infections per year [14], and serial viral infections can lead to recurrent wheezing episodes [15]. Given the concurrent time frame of recurrent HRV-induced wheezing episodes and the development of AR, it is possible that HRV infections may be one stimulus that could contribute to the initiation and progression of airway remodeling in asthma.

The human airway epithelial cell is the primary site of HRV infection and previous studies have shown that infected epithelial cells release a number of growth factors and proteins linked to airway remodeling [16–18]. The epithelium has also been suggested as a potential source of the increased numbers of mesenchymal cells in the airways during a number of disease conditions through the process of epithelial to mesenchymal transition (EMT), in which typical epithelial phenotypic proteins and morphology are lost and features of mesenchymal cells are acquired [19–22]. In the current studies, we tested the hypothesis that HRV infection of airway epithelial cells could contribute to airway remodeling by inducing EMT. Moreover, because the cytokine transforming growth factor-β (TGF-β) has been described to be a key mediator of EMT in human epithelial cells [20, 23], and is present in increased amounts in asthmatic airways [24, 25], we also examined potential interactions of HRV with TGF-β in inducing EMT.

## Methods

### Epithelial cell culture

Major group HRV species, which use intercellular adhesion molecule-1 (ICAM-1) as a receptor, do not infect more than 5-10% of primary human bronchial epithelial cells in culture [26]. Therefore, we chose to perform the current studies using the BEAS-2B human bronchial epithelial cell line, which uniformly expresses ICAM-1 [27]. This cell line was derived [28], and kindly provided by Dr. Curtis Harris (NCI, Bethesda, MD). The BEAS-2B cell line has been widely used in the literature to study EMT [23, 29–31], as well as epithelial response to HRV [18, 32–34]. BEAS-2B cells were cultured in Bronchial Epithelial Basal Medium (BEBM) (Lonza, Walkersville, MD) supplemented with bovine pituitary extract, epidermal growth factor, epinephrine, gentamicin and amphotericin B, hydrocortisone, insulin, trans-retinoic acid, transferrin, and triiodothyronine to make Bronchial Epithelial Growth Medium (BEGM) on 6-well culture plates at 37 °C and 5% CO_2_.

### HRV generation and purification

Two serotypes of HRV were used, both of which are members of the HRV-A group. Stocks of major group HRV-16, or of minor group HRV-1A, which uses members of the low-density lipoprotein receptor (LDLR) family for cell entry, were originally obtained from American Type Culture Collection (Rockville, MD). HRV-16 was propagated in WI-38 fetal lung fibroblasts cells (American Type Culture Collection, Rockville, MD), while minor group HRV-1A was propagated in H1-HeLa cells (American Type Culture Collection, Rockville, MD). Both strains of HRV were purified by sucrose density centrifugation to remove ribosomes and soluble factors as previously described [35, 36]. Viral titers were determined by using WI-38 or H1-HeLa cells grown in 96-well plates as previously described [36].

### Treatment of epithelial cell culture

When cells achieved 60% confluence, culture medium was removed and replaced with BEGM from which hydrocortisone had been removed (BEGM no HC), for 24 hours prior to stimulation. Fresh BEGM no HC was then added and BEAS-2B cells were infected with 10^4.5^ 50% tissue culture-infective dose (TCID_50_) U/ml of either purified HRV-16 or purified HRV-1A alone, 10 ng/ml recombinant TGF-β_1_ (R&D Systems, Minneapolis, MN)_,_ or the combination of each HRV and TGF-β_1,_ and subsequently incubated at 34 °C in 5% CO_2_ for specified time points. In 120-hour experiments, the medium and TGF-β_1_ were replaced at 48 and 96 hours. We demonstrated that treatment with HRV alone, TGF-β_1_ alone, or the combination, did not reduce cell viability using the 3-(4,5-dimethylthiazol-2-yl)-2,5-diphenyl tetrazolium bromide (MTT) viability assay (data not shown). For studies examining activation of mitogen activated protein kinase (MAPK) signaling pathways, BEAS-2B cells were pretreated for 24 hours in BEGM no HC then placed in BEBM for one hour prior to experiment treatment to reduce basal activation. BEBM was used for treatment medium.

### Replication-deficient HRV

To render HRV replication-deficient, stocks of purified HRV-16 and HRV-1A were exposed for 5 min to a Spectroline Model XX-15 F high intensity short wavelength (254 nm) ultra-violet (UV) lamp (Spectronics Corp., Westbury, NY) at a distance of 5 cm. It was confirmed that treated virus was replication-deficient by showing that the ability to cause lysis in appropriate host cells (WI-38 or H1-HeLa) was lost. UV-treated virus was used in experiments at doses identical to those for intact (replication competent) HRV in all experiments.

### Pharmacological Inhibitors

Commercially available pharmacological inhibitors, SB203580 and PD95089 from InvivoGen (San Diego, CA), were used to inhibit p38 MAPK and MEK1/MEK2 (ERK 1/2) MAPK pathways, respectively. For these studies, BEAS-2B cells were pretreated with the inhibitor of interest for 1 hour prior to addition of HRV-16, HRV-1A, TGF-β_1,_ and the combination of each HRV serotype with TGF-β_1_. Cells were harvested for whole-cell lysates at 24 hours.

### Whole-cell lysates

Post infection, BEAS-2B cells were lysed in ice-cold lysis buffer (1% Triton X-100 in 1 X MES buffered saline pH 7.4, containing anti-protease tablets, 50 nM sodium orthovanadate, 0.4 M sodium pyrophosphate, and 1 M sodium fluoride). Lysates were analyzed for protein content using a DC Protein Assay (BioRad Laboratories, Mississauga, ON, Canada) as manufacturer recommended.

### Western blots

Equivalent amounts of whole cell lysate were separated by 10% SDS-PAGE, and then transferred to a 0.45 μm nitrocellulose membrane. Membranes were blocked with 5% skim milk, and incubated overnight at 4 °C with the following antibodies: E-cadherin (BD Biosciences, San Jose, CA), cytokeratin-18 and cellular fibronectin (both from Sigma-Aldrich, Oakville, ON, Canada), vimentin, phospho-ERK1/2 mitogen activated protein kinase (MAPK), total-ERK1/2 MAPK, phospho-p38 MAPK, total-p38 MAPK, SLUG, SNAIL, or phospho-SMAD 2/3 (all from Cell Signaling, Danvers, MA). Membranes were then stripped and re-probed for the housekeeping protein, β-tubulin (Sigma-Aldrich, Oakville, ON, Canada).

### Densitometry analysis

Densitometry analysis was performed using ImageJ software (version 1.41, National Institute of Health, Bethesda, MD). The fold change in expression of the protein of interest was determined by normalizing the raw pixel count of the protein of interest to the raw pixel count of the respective housekeeping protein. This was then compared to the normalized value for medium control. Data is expressed as fold change over medium control unless specified otherwise.

### Assessment of morphological changes

To determine the percentage of cells exposed to each treatment that acquired a mesenchymal morphology, we used ImageJ software to measure the length and width of 75 BEAS-2B cells grown under normal medium conditions. The mean and standard deviation of the length to width ratio was then calculated for these 75 cells. We then calculated the mean length to width ratio + 10 standard deviations and established this number as a threshold that must be exceeded before a cell was considered sufficiently long and thin (spindle-like) to be classified as of a mesenchymal morphology. Using a grid reticule, we then used the same software to count a minimum of 50 cells from each treatment condition in each of three separate experiments and calculate the percentage of cells (mean ± SEM) that showed a mesenchymal phenotype in response to each treatment.

### Statistical analysis

All statistics were performed using GraphPad Prism 6 (GraphPad Software, CA). Data is presented as mean ± standard error of the mean. Non-Gaussian distributed data was analyzed by Kruskal-Wallis one-way analysis of variance (ANOVA) with Dunn’s post hoc analysis. Synergy between groups was determined by summing the values obtained in response to each individual treatment per experiment, and comparing that to the value obtained in response to the combined treatment using Wilcoxon matched-pairs analysis. For all statistical tests, a two-tailed p value of ˂0.05 was considered significant.

## Results

### Purified HRV-16 causes phenotypic changes characteristic of EMT in BEAS-2B cells, both alone and in combination with TGF-β_1_

**Fig. 1:**
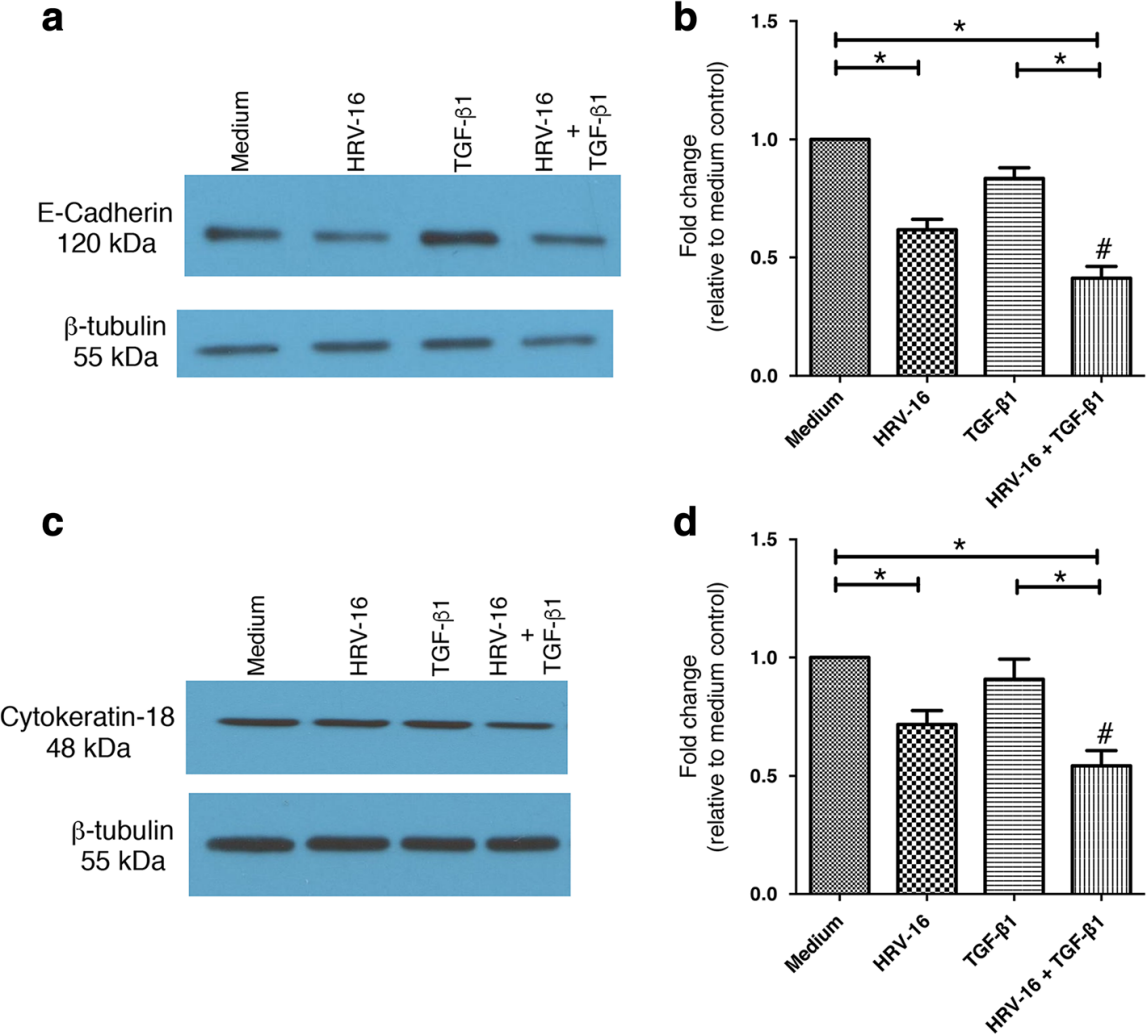
Effects of exposure for 120 h to HRV-16 alone, TGF-β_1_ alone, and the combination on expression of epithelial markers in BEAS-2B cells. BEAS-2B cells were incubated with medium alone (labeled medium in Figures), HRV-16, TGF-β_1_, and HRV-16 + TGF-β_1_ for 120 h. **a** Representative western blot for E-cadherin. **b** Densitometry analysis for E-cadherin (*n* = 11). **c** Representative western blot for Cytokeratin-18. **d** Densitometry analysis for Cytokeratin-18 (*n* = 11). Asterisk indicates significance for comparisons shown by Dunn’s post-hoc analysis after Kruskal-Wallis ANOVA. Hatchmark indicates synergy compared to the sum of responses to individual stimuli by Wilcoxon matched-pairs signed ranks test

Because it has previously been reported that 120 h of stimulation with TGF-β induces changes in epithelial phenotype consistent with EMT [23], we chose this time for our initial experiments. We initially confirmed that none of the treatments used affected cell viability under our experimental conditions, as assessed by the MTT assay (data not shown). We examined expression of the epithelial phenotypic marker proteins, E-cadherin and cytokeratin-18, as well as of the mesenchymal phenotypic marker proteins, cellular fibronectin (including EDA fibronectin) and vimentin, using western blot and densitometry. In BEAS-2B cells exposed to HRV-16, we observed a significant reduction in expression of E-cadherin (Fig. 1a and b), as well as in expression of cytokeratin-18, (Fig. 1c and d). Surprisingly, by contrast, cells exposed to TGF-β_1_ alone showed no significant reduction in the expression of either of these epithelial marker proteins. Interestingly, when cells were exposed to the combination of HRV-16 and TGF-β_1_ a striking synergistic loss of both epithelial marker proteins was observed (Fig. 1b and d).

**Fig. 2:**
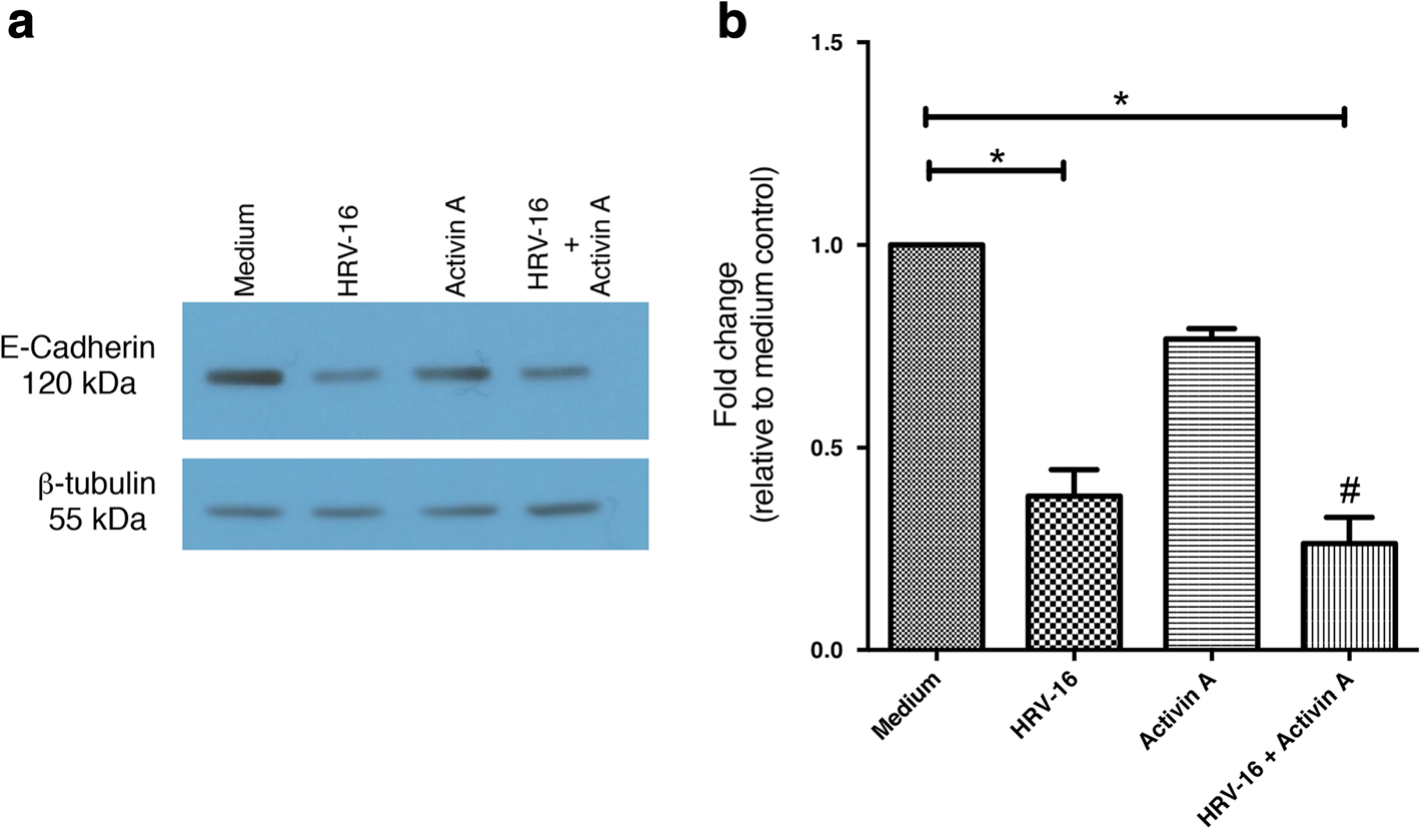
Effects of exposure for 120 h to HRV-16 alone, Activin A alone, and the combination on expression of E-cadherin in BEAS-2B cells. BEAS-2B cells were incubated with medium alone, HRV-16, Activin A, and HRV-16 + TGF-β_1_ for 120 h. **a** Representative western blot for E-cadherin. **b** Densitometry analysis for E-cadherin (*n* = 6). Asterisk indicates significance for comparisons shown by Dunn’s post-hoc analysis after Kruskal-Wallis ANOVA. Hatchmark indicates synergy compared to the sum of responses to individual stimuli by Wilcoxon matched-pairs signed ranks test

Because other studies have indicated that TGF-β_1_ alone is able to trigger the loss of epithelial markers in both BEAS-2B cells and primary human airway epithelial cells, we used another TGF receptor ligand, activin A, to examine loss of E-cadherin. Consistent with our data using TGF-β_1_, activin A also failed to significantly reduce E-cadherin expression but, like TGF-β_1_ synergistically induced loss if E-cadherin when used in combination with HRV (Fig. 2)

**Fig. 3:**
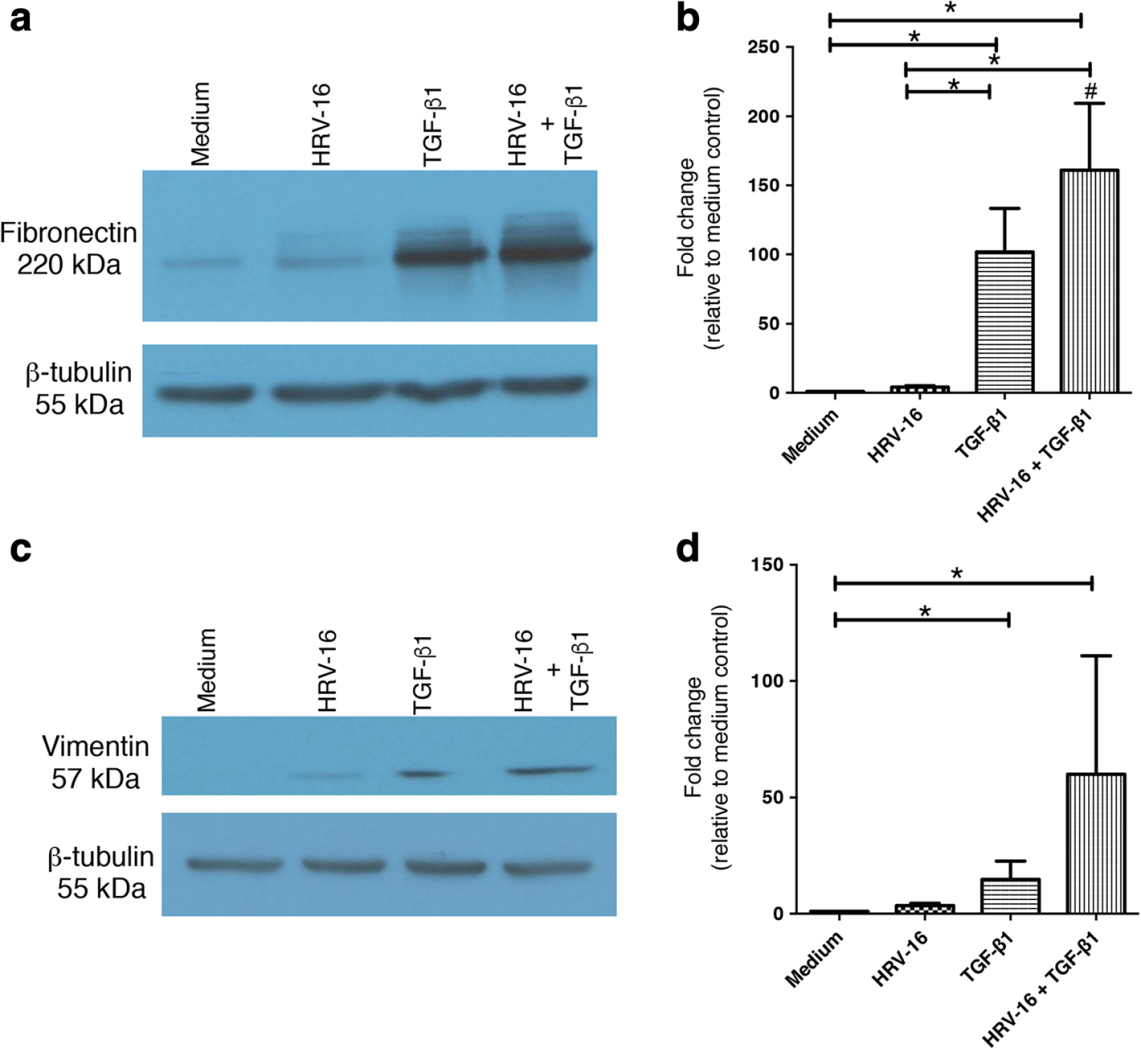
Effects of exposure for 120 h to HRV-16 alone, TGF-β_1_ alone, and the combination on expression of mesenchymal markers in BEAS-2B cells. BEAS-2B cells were stimulated with medium, HRV-16, TGF-β_1_, and HRV-16 + TGF-β_1_ for 120 h. **a** Representative western blot for fibronectin. **b** Densitometry analysis for fibronectin (*n* = 11). **c** Representative western blot for vimentin. **d** Densitometry analysis for vimentin (*n* = 11). Asterisk indicates significance for comparisons shown by Dunn’s post-hoc analysis after Kruskal-Wallis ANOVA. Hatchmark indicates synergy compared to the sum of responses to individual stimuli by Wilcoxon matched-pairs signed ranks test

Although exposure of BEAS-2B cells to HRV-16 was effective in triggering the loss of expression of epithelial marker proteins, HRV-16 alone did not significantly induce expression of the mesenchymal markers fibronectin (Fig. 3a and b), or vimentin (Fig. 3c and d). By contrast, exposure to TGF-β_1_ alone led to significant induction of both fibronectin and vimentin when compared to medium control (Fig. 3a-d). When cells were exposed to the combination of HRV-16 and TGF-β_1_ a synergistic induction of fibronectin was observed (Fig. 3b). The increase in vimentin expression was also greatest with the combination of HRV-16 and TGF-β_1_, but it did not achieve statistical significance for synergy (Fig. 3d).

**Fig. 4:**
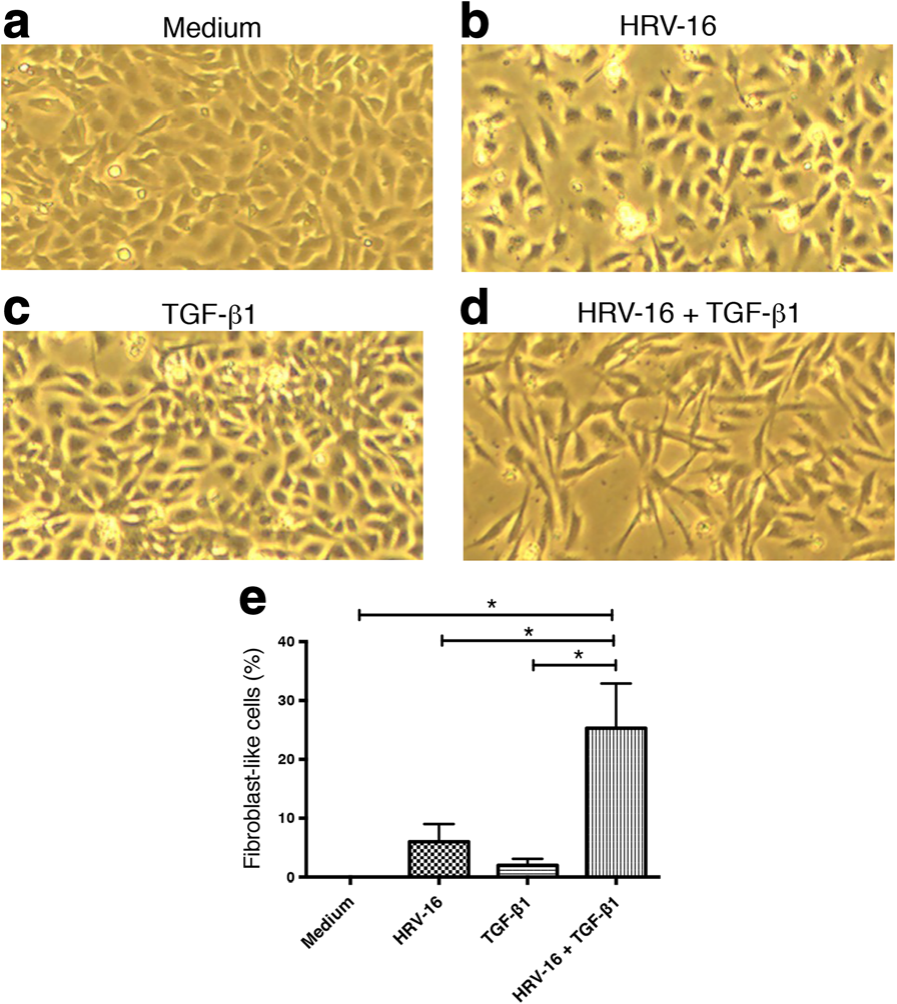
Effects of exposure for 120 h to HRV-16 alone, TGF-β_1_ alone, and the combination on morphology of BEAS-2B cells. BEAS-2B cells were stimulated with medium, HRV-16, TGF-β_1_, and HRV-16 + TGF-β_1_ for 120 h. Light microscopy images (4X magnification) showing representative morphology of cells exposed to **a** medium. **b** HRV-16 alone. **c** TGF-β_1_ alone. **d** The combination of HRV-16 + TGF-β_1_. Data are from one of 3 experiments used for quantitative analysis. **e** Quantitative analysis of percentage of cells with fibroblast-like morphology (mean ± SEM from 3 experiments). Asterisk indicates significance for comparisons shown by Dunn’s post-hoc analysis after Kruskal-Wallis ANOVA

In addition to examining the expression of phenotypic marker proteins 120 h after exposure to each stimulus alone or in combination, we also examined cellular morphology. Compared to cells cultured in medium alone, exposure to either HRV-16 alone or to TGF-β_1_ alone caused modest changes in morphology (Fig. 4a-c). Exposure to the combination of HRV-16 and TGF-β_1_, however, induced substantially more cells to adopt a spindle-like morphology consistent with that of fibroblasts or myofibroblasts (Fig. 4d). To quantify the extent of mesenchymal morphology, we calculated the mean ± standard deviation of the length to width ratio of 75 epithelial cells grown in medium. We added 10 x the standard deviation to the mean ratio and used this as a threshold to define mesenchymal morphology. We then calculated the percentage of mesenchymal cells under each experimental condition in three experiments. By definition there were no mesenchymal cells in the cells grown in medium alone. HRV-16 exposure alone caused 6 ± 3% of cells to adopt a mesenchymal morphology, while TGF-β_1_ alone induced 2 ± 1.1% of cells to become mesenchymal in shape. Neither of these was significantly increased above control. The combination of HRV-16 and TGF-β_1_, however, caused 25.3 ± 7.6% of cells to adopt a mesenchymal morphology, a significant increase above medium alone, and above each of the other two treatment groups (Fig. 4e).

### Phenotypic protein alterations occur within 24 h of exposure of BEAS-2B cells to HRV-16, or HRV-16 with TGF-β_1_

**Fig. 5:**
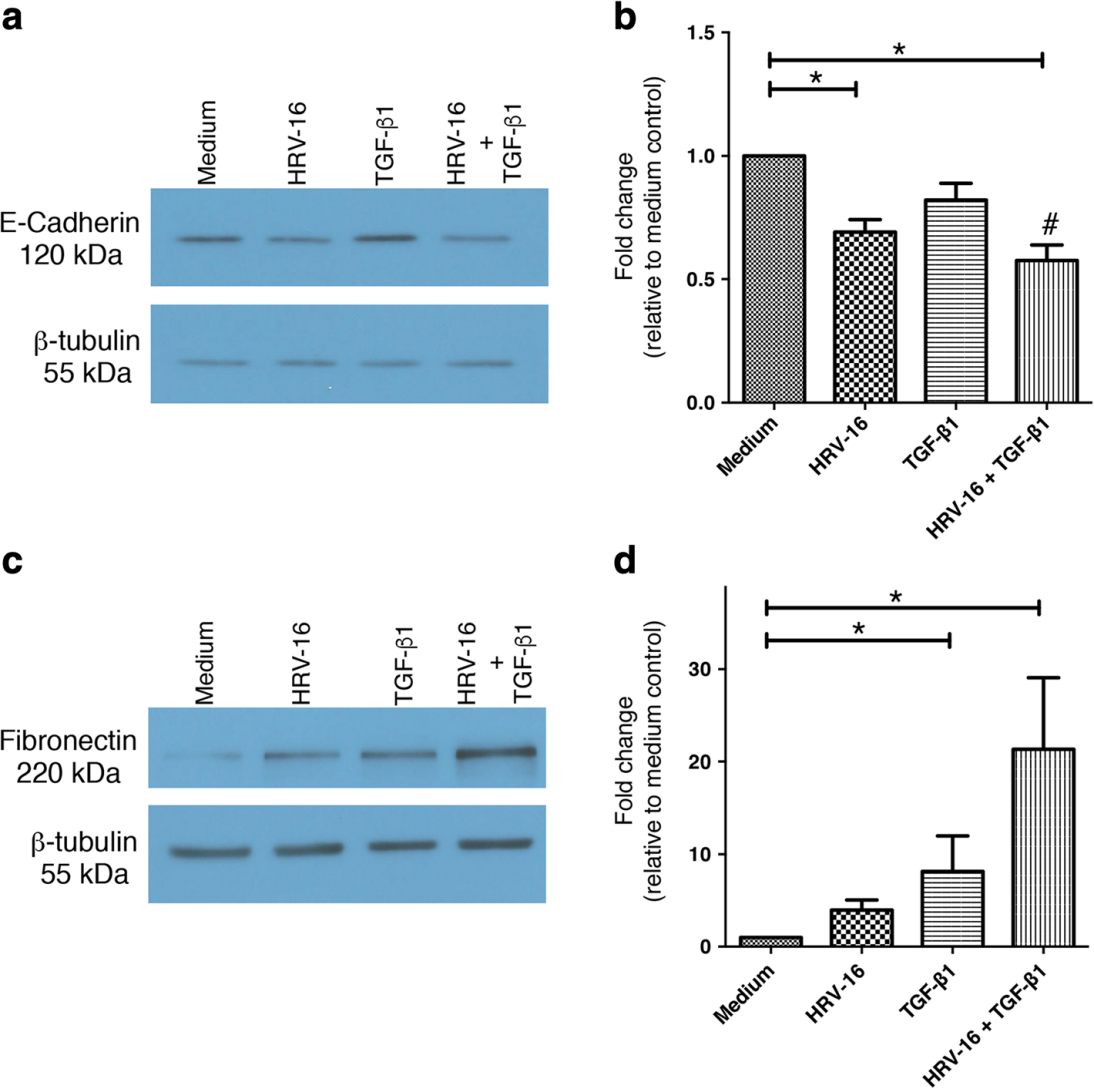
Exposure for 24 h to HRV-16 alone, TGF-β_1_ alone, and the combination alters expression of epithelial and mesenchymal markers in BEAS-2B cells. BEAS-2B cells were stimulated with medium, HRV-16, TGF-β_1_, and HRV-16 + TGF-β_1_ for 24 h. **a** Representative western blot for E-cadherin. **b** Densitometry analysis for E-cadherin (*n* = 10). **c** Representative western blot for fibronectin. **d** Densitometry analysis for fibronectin (*n* = 10). Asterisk indicates significance for comparisons shown by Dunn’s post-hoc analysis after Kruskal-Wallis ANOVA. Hatchmark indicates synergy compared to the sum of responses to individual stimuli by Wilcoxon matched-pairs signed ranks test

In order to determine how early after exposure to stimuli changes in phenotypic marker proteins could be observed, whole cell lysates of BEAS-2B cells treated with HRV-16, TGF-β_1,_ or the combination, were harvested every 24 h throughout 120 hour treatment period. Because they had given the greatest “signal-to-noise” response at 120 h, the epithelial marker, E-cadherin, and the mesenchymal marker, fibronectin, were used as readouts of EMT. Later time points are not shown because, by 24 h, BEAS-2B cells treated with HRV-16 alone or in combination with TGF-β_1_ already showed a significant reduction in expression of E-cadherin (Fig. 5a and b). Once again, treatment with the combination of HRV-16 and TGF-β_1_ induced a loss of E-cadherin expression that was synergistic compared to the sum of the responses to HRV-16 alone and TGF-β_1_ alone. Similarly, 24 h exposure to TGF-β_1_, alone or in combination with HRV-16, led to a significant induction of the mesenchymal protein fibronectin (Fig. 5c and d). Although the combination of HRV-16 and TGF-β_1_ induced the greatest expression of fibronectin, this was not synergistic compared to the sum of the responses to each stimulus alone. Morphologic changes in BEAS-2B cells began to be observed at 48 h post-treatment. The altered expression of E-cadherin and fibronectin observed within 24 h raised the possibility that early signaling events may play a role in driving observed protein and morphological alterations in BEAS-2B cells.

### Viral replication is not essential in causing changes associated with EMT

**Fig. 6:**
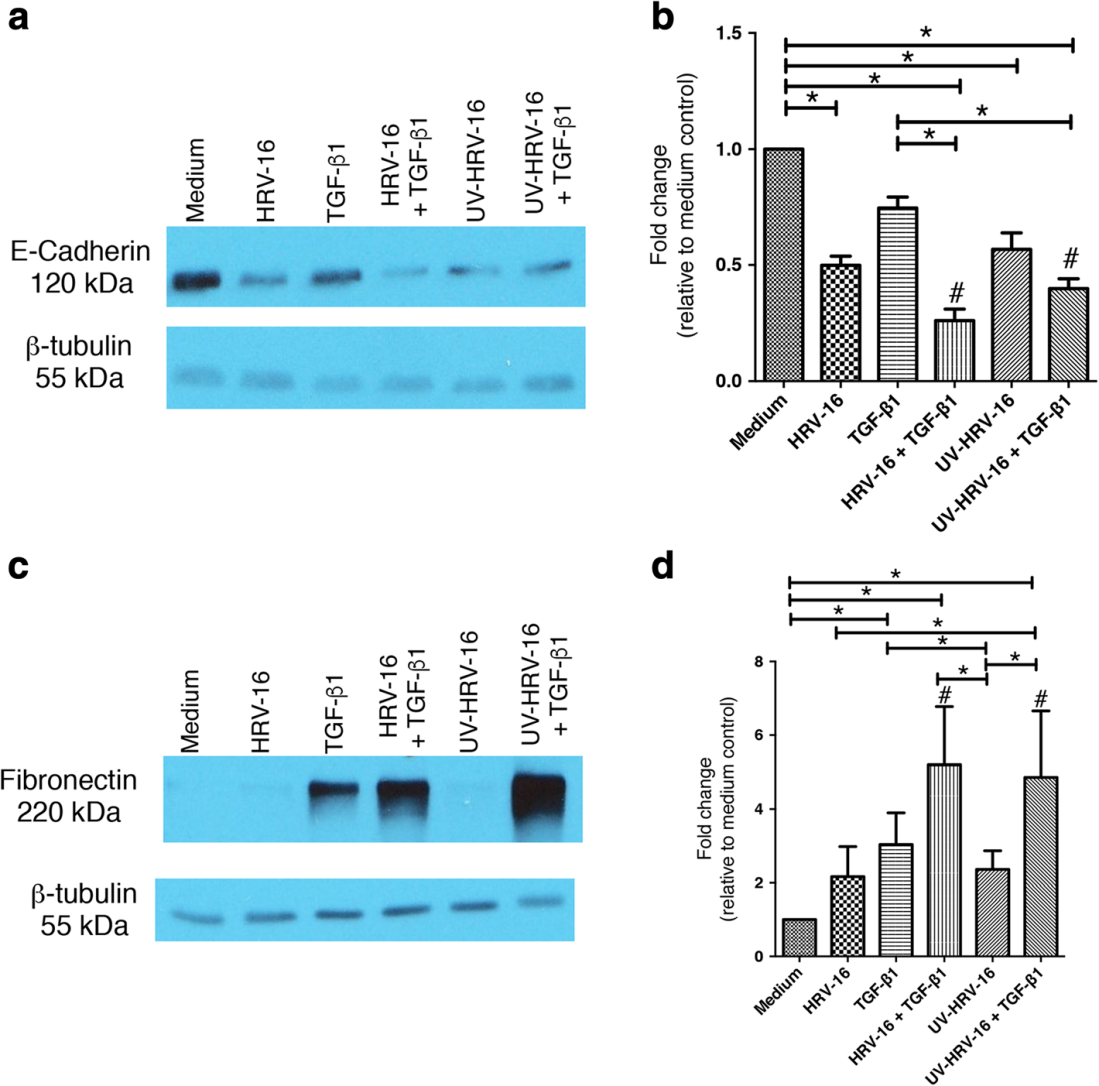
Viral replication is not essential for HRV-16 alone, or in combination with TGF-β_1_, to alter expression of epithelial and mesenchymal markers in BEAS-2B cells. BEAS-2B cells were stimulated with medium, HRV-16, TGF-β_1_, HRV-16 + TGF-β_1_, UV-HRV-16, or UV-HRV-16 + TGF-β_1_ for 120 h. **a** Representative western blot for E-cadherin. **b** Densitometry analysis for E-cadherin (*n* = 12). **c** Representative western blot for fibronectin. **d** Densitometry analysis for fibronectin (*n* = 12). Asterisk indicates significance for comparisons shown by Dunn’s post-hoc analysis after Kruskal-Wallis ANOVA. Hatchmark indicates synergy compared to the sum of responses to individual stimuli by Wilcoxon matched-pairs signed ranks test

To determine if viral replication was essential for the induction of changes consistent with EMT, BEAS-2B cells were exposed for 120 h to replication-deficient, UV-treated HRV-16, alone or in combination with TGF-β_1_. We have previously shown that virus treated in this way is still able to interact with ICAM-1, and to induce receptor-dependent signaling [37]. Treatments using intact, replication-competent, HRV-16 were included as a positive control. UV-treated HRV-16 alone, or in combination with TGF-β_1_, significantly reduced E-cadherin expression (Fig. 6a and b), with the combination treatment causing a synergistic reduction of expression compared to the sum of responses of the individual treatments. UV-treated HRV-16 alone, like intact HRV-16 alone, did not induce significant expression of the mesenchymal marker fibronectin. When used in combination with TGF-β_1_, however, UV-treated HRV-16 caused a synergistic induction of fibronectin that was comparable to that seen with intact HRV-16 + TGF-β_1_ (Fig. 6c and d). These results demonstrate that viral replication is not essential to cause phenotypic alterations characteristic of EMT, implying a potential role for viral receptor-mediated signaling.

### The minor group rhinovirus serotype, HRV-1A, causes phenotypic alterations indicative of EMT

**Fig. 7:**
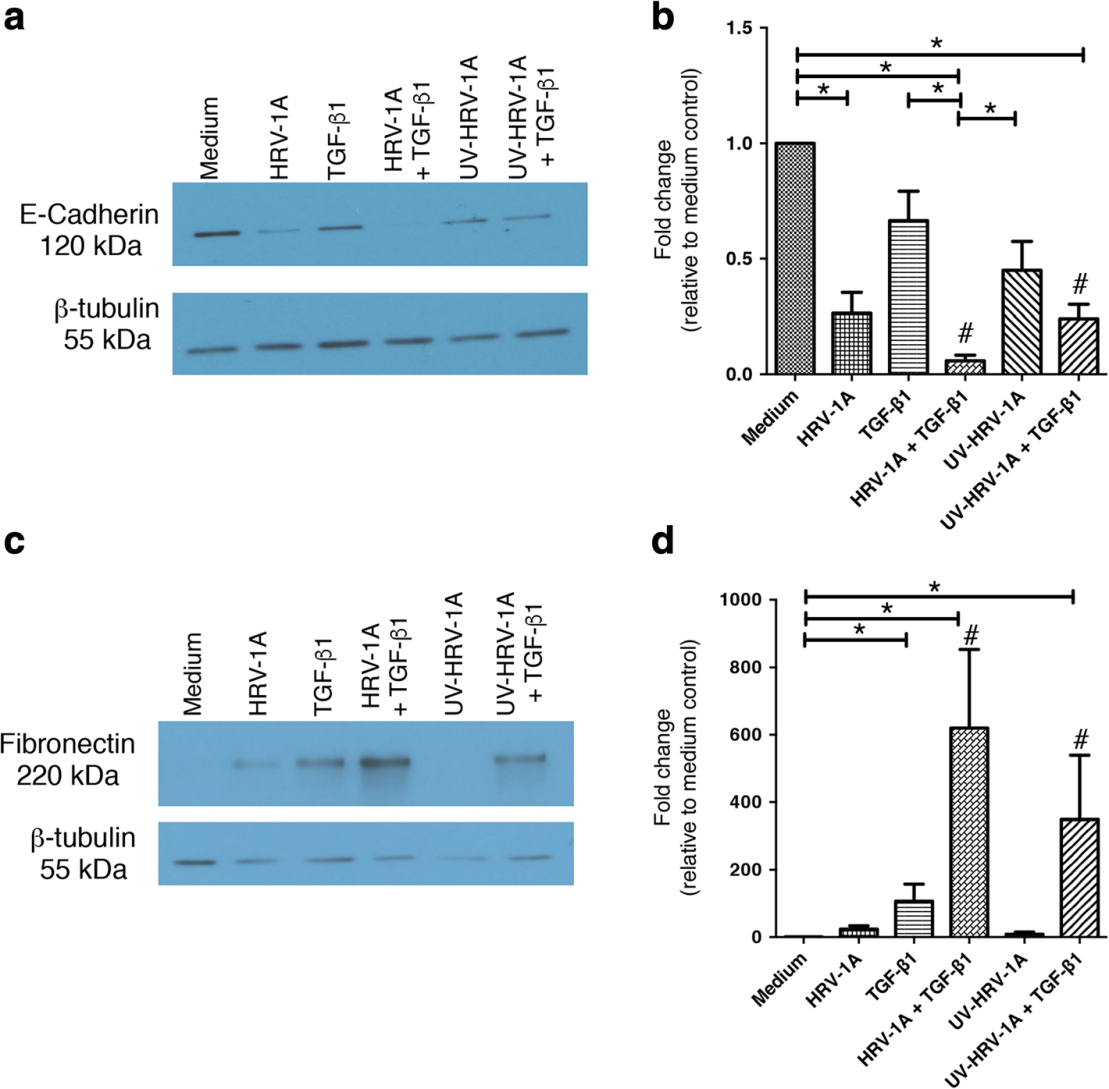
The minor group HRV-1A, alone and in combination with TGF-β_1_, also alters expression of epithelial and mesenchymal markers in BEAS-2B cells and effects do not depend upon viral replication. BEAS-2B cells were stimulated with medium, HRV-1A, TGF-β_1_, HRV-1A + TGF-β_1_, UV-HRV-1A, or UV-HRV-1A + TGF-β_1_ for 120 h. **a** Representative western blot for E-cadherin. **b** Densitometry analysis for E-cadherin (*n* = 6). **c** Representative western blot for fibronectin. **d** Densitometry analysis for fibronectin (*n* = 6). Asterisk indicates significance for comparisons shown by Dunn’s post-hoc analysis after Kruskal-Wallis ANOVA. Hatchmark indicates synergy compared to the sum of responses to individual stimuli by Wilcoxon matched-pairs signed ranks test

HRV-16 is a major group HRV, which uses ICAM-1 as its receptor. To determine if responses were selective for this group of rhinoviruses, we examined if HRV-1A, a minor group HRV that uses LDLR as its receptor, could also induce phenotypic changes consistent with EMT. BEAS-2B cells were exposed for 120 h to both replication competent and replication deficient (UV-treated) HRV-1A both alone, and in combination with TGF-β_1_. HRV-1A, both alone and in combination with TGF-β_1_, caused a significant reduction in expression of E-cadherin (Fig. 7a and b) protein expression. UV-treated HRV-1A alone caused reductions in expression of E-cadherin, but these did not achieve statistical significance. A significant reduction in E-cadherin was observed, however, for the combination of UV-HRV-1A and TGF-β_1_. In the case of both HRV-1A and UV-HRV-1A, a synergistic reduction was observed when used in combination with TGF-β_1_, compared to the sum of individual responses. Similar trends were also seen when cytokeratin-18 was examined (not shown).

As for HRV-16, HRV-1A alone was not an effective stimulus for induction of mesenchymal markers. Exposure to TGF-β_1_ alone induced significant expression of fibronectin, and this was synergistically enhanced when TGF-β_1_ was combined with either HRV-1A or UV-HRV-1A (Fig. 7c and d). Similar data were also observed when expression of vimentin was examined (not shown). Thus, both major and minor group HRV serotypes are able to induce changes consistent with EMT and, in both cases, HRV can synergize with TGF-β_1_ to trigger changes in marker proteins.

###  Role of P38 and ERK1/2 MAPK Pathways in EMT

**Fig. 8:**
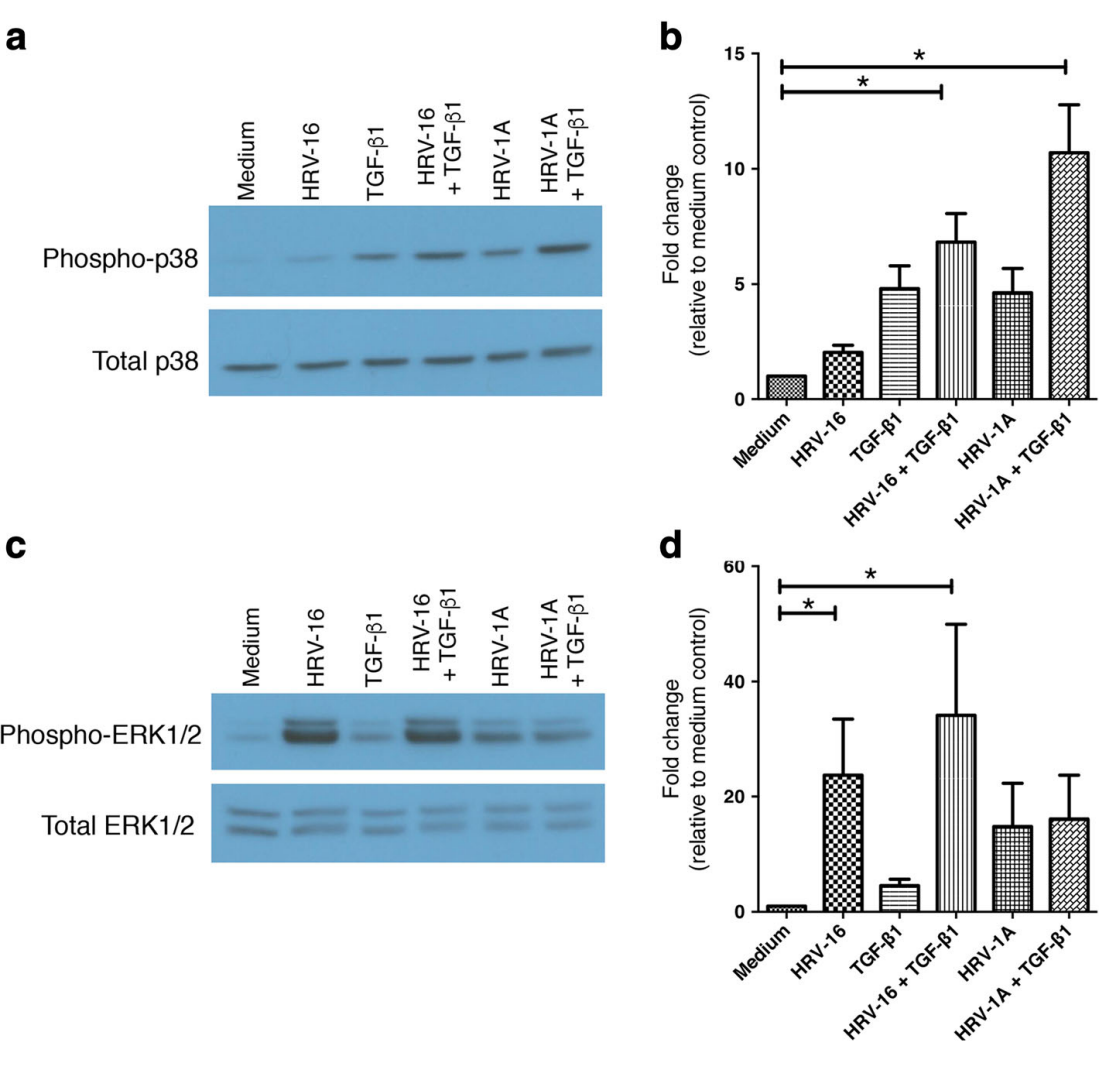
Major and minor group HRV strains, and TGF-β_1_, differentially activate MAPK signaling pathways. BEAS-2B cells were stimulated with medium, HRV-16, HRV-1A, TGF-β_1_, HRV-16 + TGF-β_1_, or HRV-1A + TGF-β_1_ for 1 h. **a** Representative western blot for phospho- and total p38. **b** Densitometry analysis for p38 (*n* = 7). **c** Representative western blot for phospho and total ERK1/2. **d** Densitometry analysis for ERK1/2 (*n* = 5). Asterisk indicates significance for comparisons shown by Dunn’s post-hoc analysis after Kruskal-Wallis ANOVA

Given that viral replication was not essential for either HRV-16 or HRV-1A to contribute to EMT, we chose to investigate early, receptor-mediated signaling pathways that may contribute to responses. Although several studies have examined early signaling pathways linked to binding of major group HRV to ICAM-1 [37–40], little is known regarding signaling of minor group HRV members via LDLR. HRV-16 interactions with ICAM-1 are known to activate both p38 and ERK1/2 MAPK signaling [37, 41, 42], so we investigated the potential role of these pathways in EMT. Initially, we examined activation of these pathways by each strain of HRV and by TGF-β_1_, alone and in combination. Although each strain of HRV alone, or TGF-β_1_ alone, induced phosphorylation of p38 MAPK (Fig. 8a and b), only the combinations of either virus strain and TGF-β_1_ led to a statistically significant activation. The response to these combinations of HRV and TGF-β_1_ appeared to be approximately additive when compared to response to each stimulus alone. TGF-β_1_ alone was ineffective in activating the ERK1/2 MAPK pathway. As previously reported, HRV-16 caused robust activation of the ERK1/2 pathway [41, 42], and this was not significantly enhanced when combined with TGF-β_1_ (Fig. 8c and d). Interestingly, HRV-1A was a less robust activator of the ERK1/2 pathway compared to HRV-16. ERK1/2 activation by HRV-1A was also not further increased in combination with TGF-β_1_.

**Fig. 9:**
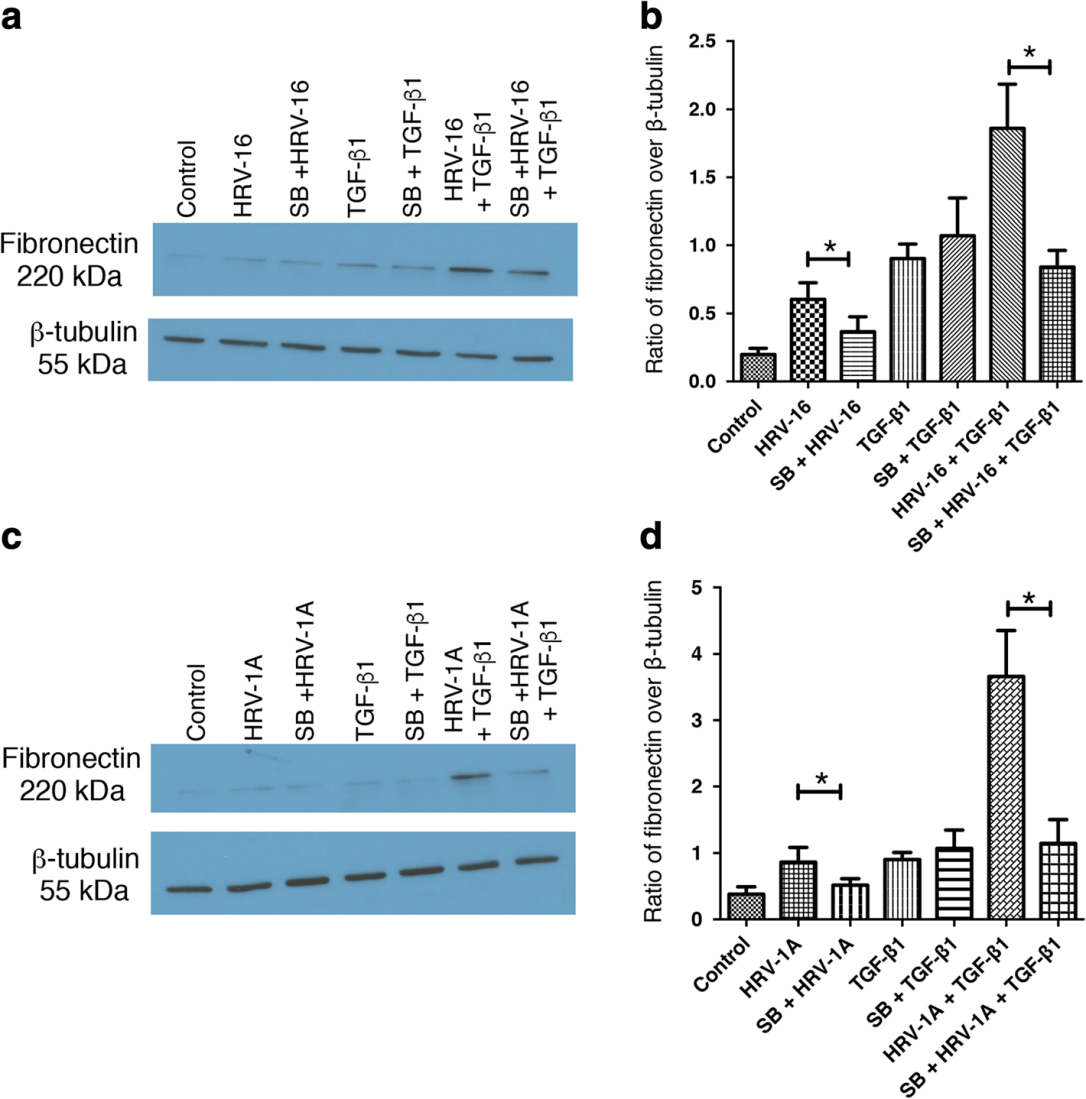
Inhibition of the p38 MAPK pathway significantly inhibits the induction of fibronectin by each HRV seroptype alone and in combination with TGF-β_1_. BEAS-2B cells were pre-treated for 1 h with the p38 MAPK pathway inhibitor, SB203580 (SB), and then stimulated with medium, HRV-16, HRV-1A, TGF-β_1_, HRV-16 + TGF-β_1_, or HRV-1A + TGF-β_1_ for 24 h. **a** Representative western blot for fibronectin using HRV-16 with and without SB. **b** Densitometry analysis for fibronectin using HRV-16 with and without SB. (*n* = 6). **c** Representative western blot for fibronectin using HRV-1A with and without SB. **d** Densitometry analysis for fibronectin using HRV-1A with and without SB. (*n* = 6). Asterisk indicates significance for comparisons shown by Dunn’s post-hoc analysis after Kruskal-Wallis ANOVA

**Fig. 10:**
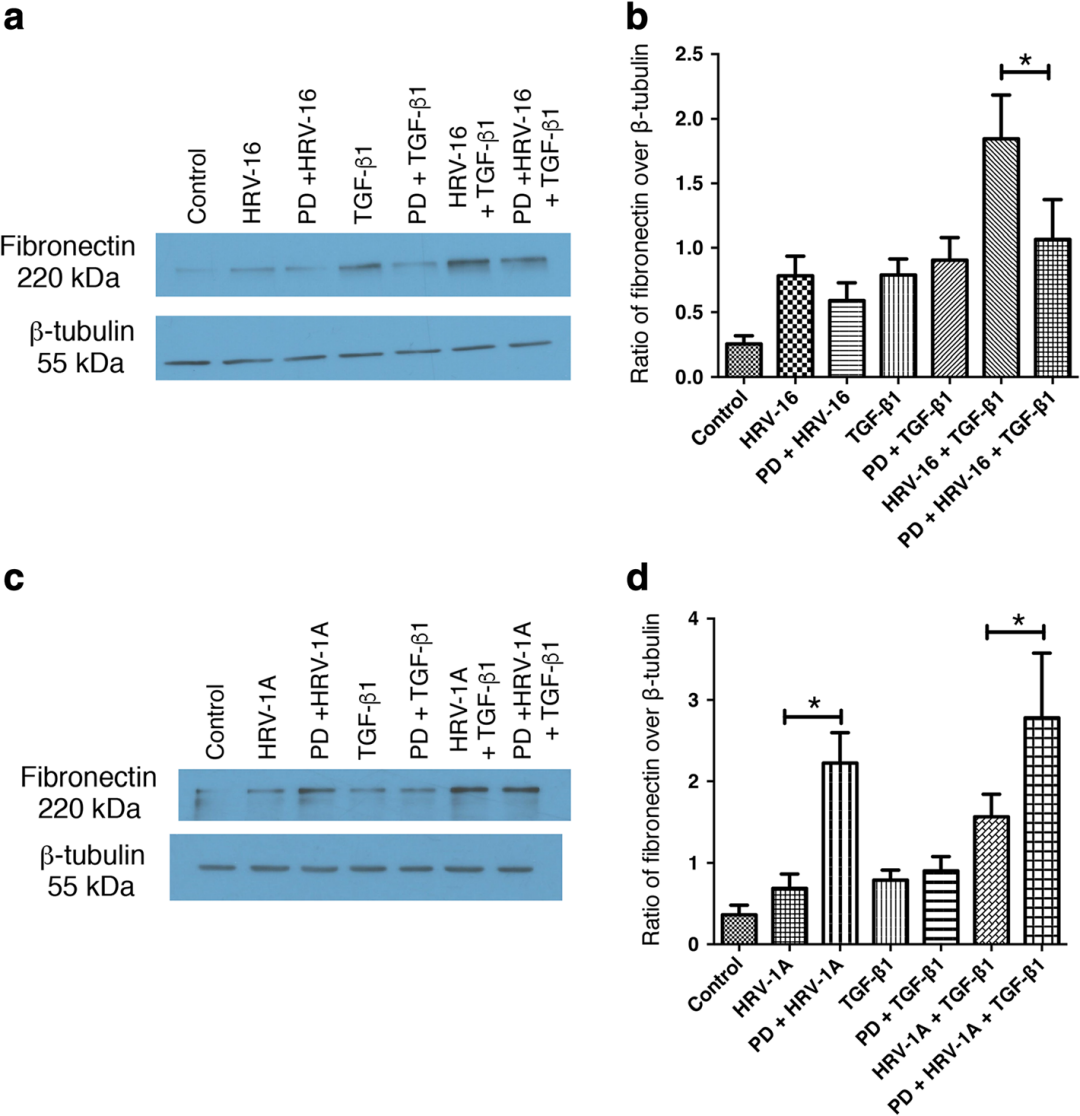
Inhibition of the ERK/1/2 MAPK pathway differentially affects the induction of fibronectin by the combination of each HRV serotype with TGF-β_1_. BEAS-2B cells were pre-treated for 1 h with the ERK1/2 MAPK pathway inhibitor, PD98059 (PD), and then stimulated with medium, HRV-16, HRV-1A, TGF-β_1_, HRV-16 + TGF-β_1_, or HRV-1A + TGF-β_1_ for 24 h. **a** Representative western blot for fibronectin using HRV-16 with and without PD. **b** Densitometry analysis for fibronectin using HRV-16 with and without PD. (*n* = 6). **c** Representative western blot for fibronectin using HRV-1A with and without PD. **d** Densitometry analysis for fibronectin using HRV-1A with and without PD. (*n* = 6). Asterisk indicates significance for comparisons shown by Dunn’s post-hoc analysis after Kruskal-Wallis ANOVA

To determine if activation of these MAPK pathways may contribute to EMT-like changes in BEAS-2B cells, we used the pharmacological inhibitors SB203580 and PD98059 to inhibit the p38 and ERK 1/2 MAPK pathways, respectively. Given that morphological changes in BEAS-2B did not begin to be observed until 48 h after treatments, we examined expression of phenotypic marker proteins as an outcome measure. Because fibronectin gave the largest signals in our earlier experiments, we selected this protein as the read out of phenotypic changes. The p38 pathway inhibitor, SB203580, significantly decreased the induction of fibronectin expression by HRV-16 alone, HRV-1A alone, and most strikingly, by the combination of each virus with TGF-β_1_ (Fig. 9a-d). Blocking the ERK1/2 MAPK pathway using PD95089 significantly inhibited the induction of fibronectin by the combination of HRV-16 and TGF-β_1_, while having little to no effect on the modest responses to either stimulus alone (Fig. 10a and b). Conversely, PD98059 enhanced fibronectin induction in response to HRV-1A alone or in combination with TGF-β_1_ (Fig. 10c and d).

### HRV and TGF-β_1_ both induce expression of the E-cadherin transcriptional repressor, SLUG

**Fig. 11:**
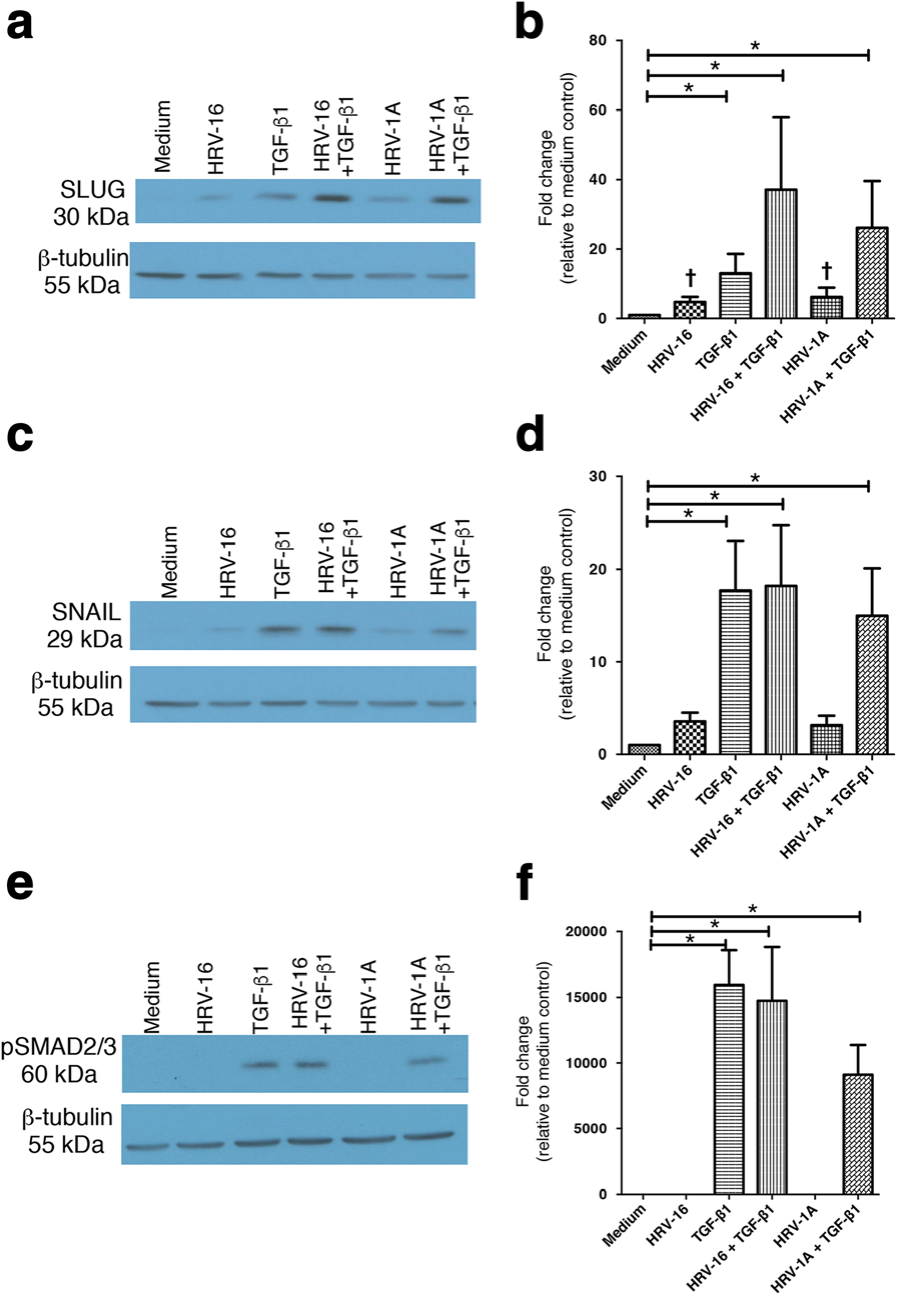
Activation of the transcriptional repressor SLUG, but not the SNAIL or Smad2/3 pathways may contribute to EMT induced by the combination of HRV and TGF-β_1_. BEAS-2B cells were stimulated with medium, HRV-16, HRV-1A, TGF-β_1_, HRV-16 + TGF-β_1_, or HRV-1A + TGF-β_1_ for 6 h. **a** Representative western blot for SLUG. **b** Densitometry analysis for SLUG (*n* = 8). **c** Representative western blot for SNAIL. **d** Densitometry analysis for SNAIL (*n* = 6). **e** Representative western blot for pSMAD2/3. **f** Densitometry analysis for pSMAD2/3 (*n* = 6). Asterisk indicates significance for comparisons shown by Dunn’s post-hoc analysis after Kruskal-Wallis ANOVA. † Indicates *p* < 0.02 compared to medium control stimuli by Wilcoxon matched-pairs signed ranks test

Given the ability of HRV-16 and HRV-1A to synergize with TGF-β1 to cause phenotypic changes characteristic of EMT in BEAS-2B cells, we examined whether HRV could modulate known signaling pathways linked to TGF-β_1_ receptor signaling. We examined modulation of the transcription factors, SLUG and SNAIL, as well as activation of SMAD2/3. BEAS-2B cells were stimulated with HRV-16 alone, TGF-β_1_ alone, HRV-16 + TGF-β_1_, HRV-1A alone, and HRV-1A + TGF-β_1_ for 6 hours and whole cell lysates were analyzed. Although induction of SLUG by HRV-16 alone or HRV-1A alone did not achieve statistical significance by post-hoc analysis after ANOVA, both HRV serotypes induce significantly (p <0.02 in each case) increased in SLUG expression compared to control by appropriate paired analyses. As expected, TGF-β_1_ alone also induced expression of SLUG, but expression was markedly increased in response to the combinations of HRV-16 + TGF-β_1_ and HRV-1A + TGF-β_1_ to levels that were at least additive compared to responses to individual stimuli (Fig. 11a and b). As expected, TGF-β_1_ also induced expression of SNAIL. By contrast, neither HRV-16 alone or HRV-1A alone induced significant expression of SNAIL and the combination of each strain of HRV with TGF-β_1_ caused no additional activation above that seen with TGF-β_1_ alone (Fig. 11c and d). Similarly, neither HRV-16 alone nor HRV1A-alone induced phosphorylation of SMAD2/3, and the combination of either HRV serotype with TGF-β_1_ caused no activation above that observed with TGF-β_1_ alone (Fig. 11e and f).

## Discussion

Recurrent HRV-induced wheezing illnesses in the first 3 years of life are a major risk factor for subsequent development of asthma [13]. Given that this time frame also coincides with the development of airway remodeling, we hypothesized that HRV infections may contribute to aspects of airway remodeling by altering epithelial cell biology. We, and others, have previously shown that HRV infection of human airway epithelial cells, both *in vitro* and in *vivo*, can lead to the induction of a number of growth factors linked to airway remodeling [16–18], as well as of chemotactic agents that can cause migration of mesenchymal cells towards the epithelial layer [43]. We now provide direct evidence that two different serotypes of HRV are also able to drive epithelial phenotypic and morphological changes that are indicative of EMT, further supporting a potential role of HRV infections in airway remodeling. Our data contrast somewhat with a recent report that HRV-39 could induce EMT-like marker protein changes in regenerating, but not normal epithelial cell cultures [44].

The pleiotropic cytokine, TGF-β_1_ has been reported to be a key initiator of EMT in profibrotic processes occurring in the airways in asthma [20, 23, 45], and levels of TGF-β_1_ are elevated in the airways of both children and adults with asthma [24, 25]. Given the central role ascribed to TGF-β_1_ in triggering EMT, we were surprised to observe that both serotypes of HRV tested were more effective than TGF-β_1_ at inducing the loss of the epithelial phenotypic marker proteins, E-cadherin and cytokeratin-18. This data contrasts with prior reports of TGF-β_1_ triggering the loss of epithelial markers in both BEAS-2B cells [23] and primary human airway epithelial cells [20]. The reasons for this discrepancy are unclear, although differences in culture medium and growth conditions could potentially explain the difference between our data and those of Doerner and colleagues who grew BEAS-2B cells in a keratinocyte culture medium [23]. It is not feasible that BEAS-2B cells used in our studies do not express receptors for TGF-β_1_, given that this cytokine was clearly effective in inducing mesenchymal markers. Our data were consistent with those of Heijink and coworkers who also found that TGF-β_1_ had only a modest effect on the loss of epithelial markers [46]. Nonetheless, to examine this issue further we used activin A, another member of the TGF-β superfamily that we have previously shown to be induced in both BEAS-2B and primary airway epithelial cells in response to HRV infection [16] as an alternative ligand for the TGF-β receptor. Consistent with the data obtained using TGF-β_1_, activin A also caused only a modest loss of E-cadherin but synergized with HRV-16 in inducing loss of this epithelial marker.

It is not clear why exposure to HRV is so effective in downregulating epithelial markers. One possible explanation relates to observations that, in addition to triggering some of the signal pathways studied in the current work, both the major (ICAM-1) and minor (LDL-receptor) HRV receptors are known to be associated with lipid rafts [47], which, in turn, are known to associated with the actin cytoskeleton [48]. Indeed, it has been known for some time that ICAM-1 is associated with the actin cytoskeleton [49]. More recently, it has been shown that binding of HRV to ICAM-1 is known to recruit the cytoskeletal linker protein ezrin to ICAM-1 [37]. Thus binding of HRV to it receptor (ICAM-1 or LDLR) likely leads to rearrangements in the actin cytoskeleton and/or microtubules, as has been shown for other picornaviruses [50]. Since the actin cytoskeleton is integral to the retention of an epithelial phenotype, and since cytokeratin and junctional proteins, such as E-cadherin, are closely affiliated with the actin cytoskeleton, this may lead to an increased capacity of HRV to induce loss of these epithelial markers, setting the stage for mesenchymal transition.

In contrast to data for epithelial markers, TGF-β_1_ alone was more effective than either serotype of HRV alone in inducing expression of the mesenchymal markers, fibronectin and vimentin. There was a clear interaction between the two stimuli, however, as the combination of HRV and TGF-β_1_ clearly induced synergistic changes in both epithelial and mesenchymal markers. The combination of HRV and TGF-β_1_ was also most effective in causing morphological changes consistent with EMT. This is the first demonstration of an interaction between HRV and TGF-β_1_ in causing EMT-like changes, but our data show some similarities to those of Heijink and co-workers, who also found that TGF-β_1_ had only modest effects on reducing expression of epithelial markers, but synergized with house dust mite to induce changes consistent with EMT [46]. Moreover, TGF-β_1_ has also been reported to synergize with nicotine to induce EMT [51].

EMT is generally considered to be a slow and progressive process, with intermediate stages in which cells have the characteristics of both epithelial and mesenchymal cells [52]. It was unexpected, therefore, to find that changes associated with EMT, and interactions with TGF-β_1_, could already be observed within 24 h of HRV exposure, and to find that viral replication was not required for HRV to exert its effects. This implied a role for early, receptor-dependent signaling, rather than replication dependent signaling through double stranded RNA pattern recognition receptors. Given that both major and minor group HRV serotypes induced phenotypic changes, relevant signals must be sent via either ICAM-1 or LDLR. Because little is known about early signaling through LDLR, we examined activation of the p38 and ERK1/2 MAPK pathways, as these are known to be activated both by TGF-β_1_ [53, 54], and via ICAM-1 signaling [37, 41, 42], to determine if these may also contribute to signaling via the LDLR. As expected, HRV-16 and TGF-β_1_ each induced activation of the p38 MAPK pathway and we also provide the first evidence that HRV-1A also induced p38 activation. The combination of each serotype of HRV with TGF-β_1_ led to an approximately additive activation in each case. Despite a prior report of ERK1/2 activation by TGF-β_1_ in intestinal epithelial cells [54], TGF-β_1_ alone did not induce appreciable activation of the ERK1/2 pathway in BEAS-2B cells. As expected, HRV-16 induced robust activation of ERK1/2 and this was slightly enhanced in the presence of TGF-β_1_. By contrast, HRV-1A was considerably less effective in activation of ERK1/2 and there was no additional response in the presence of TGF-β_1_.

To determine the potential role of MAPK pathways in EMT induced by HRV and TGF-β_1_, selective inhibitors of each pathway were used to examine effects on fibronectin expression. Inhibition of the p38 pathway significantly inhibited fibronectin expression induced by either serotype of HRV alone, and in combination with TGF-β_1_. Surprisingly, inhibition of this pathway had little effect on responses to TGF-β_1_ alone. Although TGF-β_1_ did not induce a marked activation of ERK1/2, inhibition of this pathway significantly reduced fibronectin induction in response to the combination of HRV-16 and TGF-β_1_ without affecting the modest response to HRV-16 alone. Interestingly, fibronectin induction in response to HRV-1A, either alone or combined with TGF-β_1_, was enhanced by inhibition of the ERK1/2 pathway. Thus the p38 MAPK pathway appears to play a role in induction of EMT by both major and minor group HRV strains, alone and in conjunction with TGF-β_1_, but the ERK1/2 pathway exerts different effects depending upon the HRV strain receptor involved.

To further examine the underlying basis of the potential mechanisms by which HRV may interact with TGF-β_1_ to induce EMT, we examined pathways implicated in TGF-β_1_ signaling. We provide the first evidence that both HRV-16 and HRV-1A are not only able to induce expression of the E-cadherin transcriptional repressor, SLUG, but that the combination of each HRV serotype with TGF-β_1_ induces activation of SLUG that is greater than that seen with TGF-β_1_ alone. This provides a potential mechanism to support the reduced expression of E-cadherin seen with HRV alone, and the synergistic induction observed with the combination of HRV and TGF-β_1_. Interestingly, consistent with an earlier report [44], while each HRV serotype appeared to modestly induce activation of a second, related transcriptional repressor SNAIL, the combination of HRV and TGF-β_1_ did not induce any activation above that observed with TGF-β_1_ alone. Finally, we showed that neither HRV-16 nor HRV-1A induced activation of the SMAD2/3 pathway, and the combination of either strain of virus with TGF-β_1_ had no effect above that seen with TGF-β_1_ alone.

A limitation of this study is the use of the BEAS-2B bronchial epithelial cell line. This was chosen because less than 10% of primary cells can be infected by major group HRV strains [26], raising concerns that insufficient “signal-to-noise” would be obtained. As noted, the BEAS-2B cell has been commonly used to examine EMT [23, 29–31], and has been shown to reproduce effects observed with HRV in primary cells in a number of studies [16, 55–57]. Nonetheless, it will be important to extend these studies to primary epithelial cells. This should be feasible having established that HRV-1A induced EMT as minor group HRV can infect a greater percentage of primary cells than major group strains [58]. It will also be important to determine if HRV-induced EMT is enhanced in epithelial cells from asthmatic children or adults.

It should be noted that, while EMT is a well-recognized process in both physiological and pathophysiological conditions, it remains unclear what percentage of fibroblasts and myofibroblasts in the asthmatic airways derive from EMT. Studies using genetic fate tracking of lung epithelial cells in a mouse model of allergic asthma reported that approximately 30% of murine lung fibroblasts were derived from epithelial cells that underwent EMT [59], but it is far from clear if this is the case in humans.

## Conclusions

In summary, we provide the first evidence that both HRV-16 and HRV-1A, representing major group and minor group serotypes, respectively, are able to induce phenotypic and morphological changes in bronchial epithelial cells that are consistent with EMT. HRV appears to play a greater role in the loss of epithelial markers, but both serotypes of HRV synergize with TGF-β_1_ to induce changes in epithelial and mesenchymal markers and to change morphology. The effects of HRV on EMT do not depend on viral replication but appear to involve activation of MAPK pathways, as well as activation of the transcriptional repressor SLUG. Taken together, our data add further support for the concept that repeated HRV infections may contribute to the development and progression of airway remodeling in asthma.
